# Multiple Endocrine Neoplasia Type 1 Syndrome: A Case Report and Review of Literature

**DOI:** 10.7759/cureus.12073

**Published:** 2020-12-14

**Authors:** Hiya Boro, Suraj Kubihal, Saurabh Arora, Vijay Kubihal, Nikhil Tandon

**Affiliations:** 1 Endocrinology, Diabetes, and Metabolism, All India Institute of Medical Sciences, New Delhi, IND; 2 Nuclear Medicine, All India Institute of Medical Sciences, New Delhi, IND; 3 Radiology, All India Institute of Medical Sciences, New Delhi, IND; 4 Endocrinology and Metabolism, All India Institute of Medical Sciences, New Delhi, IND

**Keywords:** parathyroid adenoma, insulinoma, pancreatic net, pituitary adenoma

## Abstract

Multiple endocrine neoplasia type 1 (MEN1) or Wermer’s syndrome is a genetic disease characterized by involvement of multiple endocrine glands, primarily involving parathyroid, pancreas, and pituitary. Other additional features include foregut carcinoids; non-functioning adrenal tumors; and skin lesions such as lipomas, collagenomas, and angiofibromas. Here, we describe our experience in managing a patient who presented to us with spontaneous episodes of hypoglycemia and was diagnosed with insulinoma. Detailed clinical and biochemical evaluation unraveled the diagnosis of MEN1 in the patient and her family members who constituted a large kindred. This case highlights the importance for evaluation of MEN1 in a patient or his/her family members in the setting of clinical and biochemical suspicion. In addition, we have also discussed the utility of the latest diagnostic and therapeutic modalities for management of MEN1.

## Introduction

Multiple endocrine neoplasia type 1 (MEN1) or Wermer’s syndrome is a genetic disease caused by inactivating mutation of MEN1 gene. It is characterized by parathyroid, pituitary, and pancreatic neoplasms. In addition, other features include foregut carcinoids; non-functioning adrenal tumors; skin lesions such as lipomas, angiofibromas, and collagenomas. Most of the mutations are germline, but sporadic and de novo mutations can also occur. The disease affects all age groups from five to 80 years. Most of the manifestations are evident by the fifth decade of life. The clinical manifestations of MEN1 are related to the tumor site and the hormonal products released. In the absence of treatment, mortality rate is 50% by 50 years of age [1].

Here, we describe our experience with a case of MEN1 syndrome who had presented with one predominant organ-related symptom. Subsequently, on evaluation she was found to harbor multiple tumors. She was the index case of a large kindred, and her diagnosis brought to attention the presence of MEN1 in other family members.

## Case presentation

A 46-year-old female presented to us with multiple episodes of increased hunger, diaphoresis, palpitations, tremors, anxiety, and dizziness for the last eight months. These episodes usually occurred in the fasting state. During one such episode, her blood glucose recorded by glucometer was 35 mg/dl. Her symptoms were reversed by intake of carbohydrate diet. Though Whipple’s triad was not established as plasma glucose was not measured, the symptomatology did merit for further evaluation [2]. She had no history of diabetes, chronic liver disease, chronic kidney disease, or history of intake of any drug known to cause hypoglycemia.

**Figure 1:**
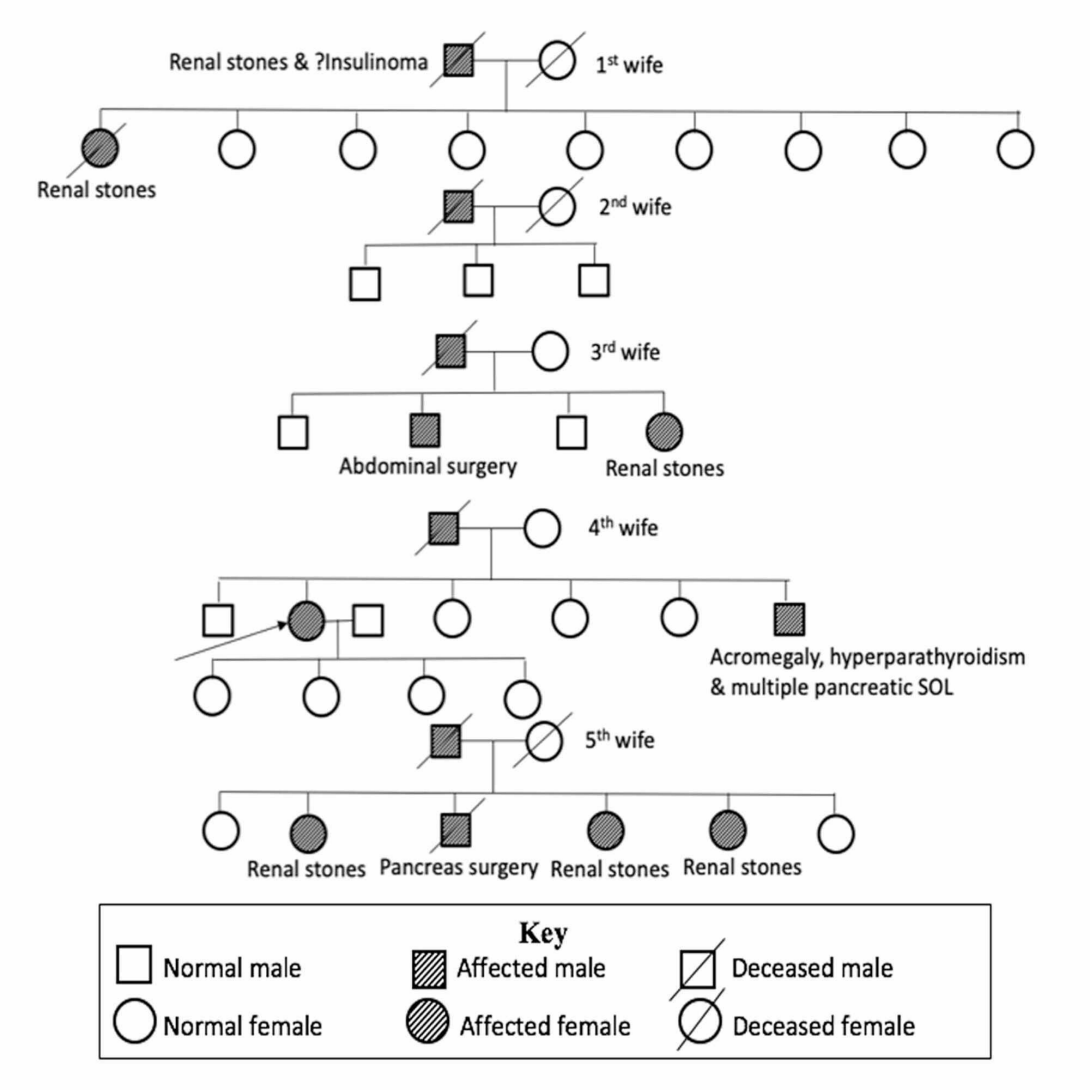
Pedigree chart of our patient SOL, Space-occupying lesion; (?) insulinoma-suspected insulinoma

On further probing, she revealed history of recurrent renal stones for the past 16 years. In addition, she had history of galactorrhea and oligomenorrhea for the past two years. Apart from this, there was no other significant history. She was the index case in her family. However, her family history did reveal that she was part of a large kindred. She had five siblings and 22 step siblings from the five marriages of her father. Patient’s father who had similar episodes of neuroglycopenic symptoms [suspected (?) insulinoma] and history of renal stones (Figure 1) had expired before evaluation. There was history suggestive of at least one MEN1-associated endocrine tumor among eight of her siblings. The pedigree chart is shown in Figure 1.

After admission in our ward, the patient had an episode of spontaneous hypoglycemia, and critical sample was analyzed that revealed non-ketotic hyperinsulinemic hypoglycemia (blood glucose of 40 mg/dl with corresponding serum insulin 19.4 µU/ml and C peptide of 4.9 ng/ml, blood ketones of 0.1 mmol/L) [3]. Serum insulin and C peptide were measured by electrochemiluminescence (ECLIA) assay (Cobas e411, Roche diagnostics, Germany), while blood ketones were measured on a finger prick test using a point of care device (normal range 0.4-0.5 mmol/L). Patient also had parathyroid hormone (PTH)-dependent hypercalcemia with hypercalciuria [serum total calcium 12.3 mg/dl (normal range: 8.5-10.5 mg/dl), serum intact PTH of 210 pg/ml (normal range 15-65 pg/ml), 24-hour urinary calcium of 450 mg/day (normal range < 250 mg/day)]. Serum calcium was measured on Roche Hitachi 917 (automated analyzer), while serum intact PTH was measured on ECLIA (minimum detection limit 1.5 pg/ml). Her serum prolactin level, measured by ECLIA, was also elevated, 92.6 ng/ml (normal range, 10-29 ng/ml). Other pituitary hormones were within normal limits.

A suspicion of MEN1 syndrome was made, which was later confirmed by positive screening test for MEN1 gene mutation (Deletion: c.824-832delGGTACCCCA in exon 5 of MEN1 gene on whole gene sequencing).

**Figure 2:**
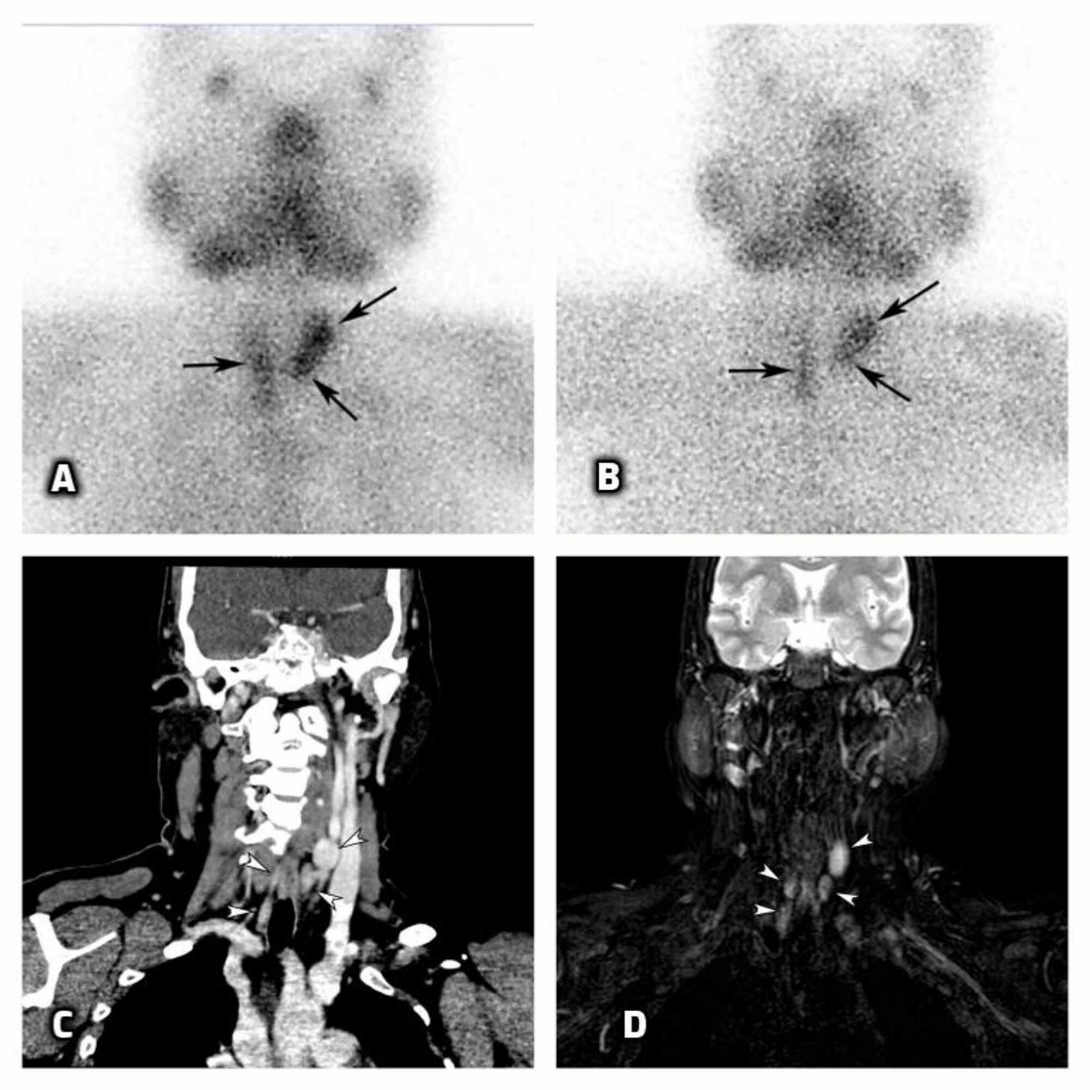
Technetium-99m SESTAMIBI (A, B), 4D CT (C), and 4D MRI (D) scans of the patient showing parathyroid adenomas Early (A) and delayed (B) phase images of SESTAMIBI scan showing elongated areas of early increased tracer uptake in right inferior, left superior, and left inferior parathyroid gland, persisting in delayed phase. Coronal venous phase image of 4D CT (C) and coronal T2-weighted 4D MRI image (D) showing involvement of all four parathyroid glands.

For evaluation of PTH-dependent hypercalcemia, she underwent technetium-99m SESTAMIBI scan that revealed involvement of three parathyroid glands (left superior, left inferior, and right paratracheal) (Figures 2A, 2B). However, the four-dimensional computed tomography (4D CT) (Figure 2C) and four-dimensional magnetic resonance imaging (4D MRI) (Figure 2D) showed involvement of all the four glands.

**Figure 3:**
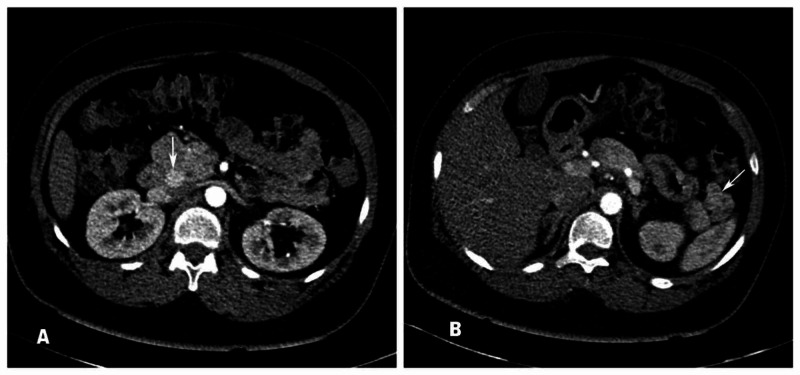
CT images showing pancreatic NET Axial pancreatic phase images of multiphase contrast-enhanced CT images show hyper-enhancing nodule in the pancreatic head (A) and another heterogeneous exophytic lesion with eccentric nodular hyperenhancement in the pancreatic tail region (B) s/o pancreatic neuroendocrine tumor (NET).

For evaluation of hyperinsulinemic hypoglycemia, she underwent multiphase contrast-enhanced computed tomography (CT) of abdomen that revealed two mass lesions in the pancreatic head and tail, respectively (Figures 3A, 3B).

**Figure 4:**
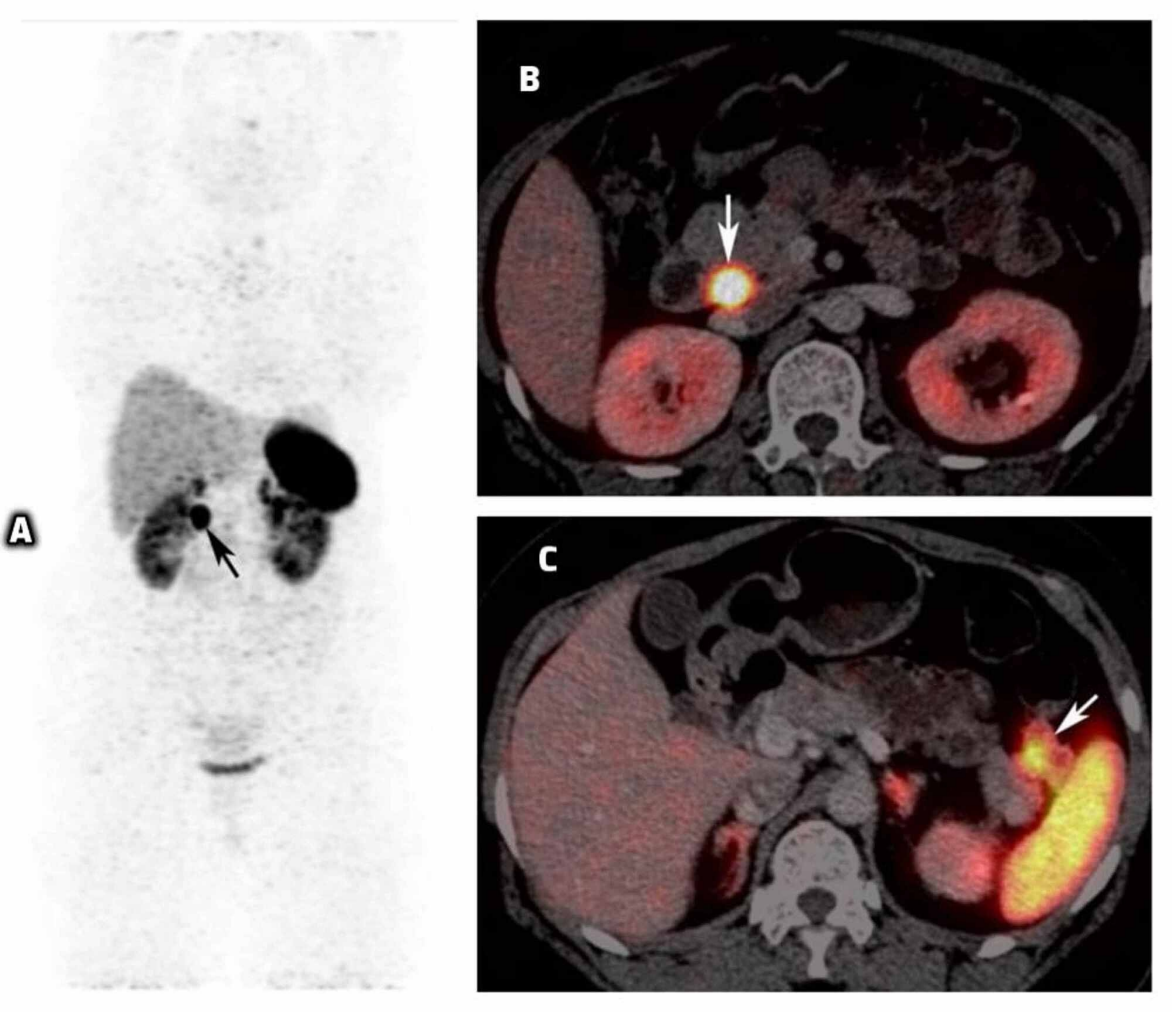
Ga-68-DOTANOC scan images of the patient Maximum intensity projection (MIP) image (A) and axial fused positron emission tomography/computed tomography (PET/CT) images (B and C) show two somatostatin receptor expressing lesions in the pancreatic head (1.6 cm x 1.5 cm) (B) and tail region (3.2 cm x 2.2 cm) (C), respectively.

Patient also underwent Ga-68-DOTANOC positron emission tomography (PET)/computed tomography (CT) that revealed somatostatin receptor (SSTR) expressing lesions in the head of the pancreas (1.6 cm x 1.5 cm) and tail of the pancreas (3.2 cm x 2.2 cm) (Figures 4A-4C). She also underwent endoscopic ultrasonography (EUS) that revealed multiple hypoechoic round lesions in the pancreas (max: 3.5 cm x 2.1 cm x 0.8 cm) with vascularity in the lesions, two lesions of diameter 2.1 cm and 1.1 cm in the body of pancreas, and four small lesions in the tail of pancreas (~8 mm each).

**Figure 5:**
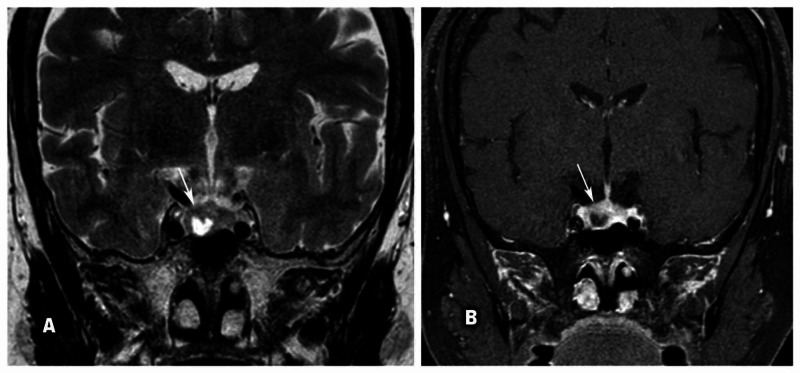
MRI images showing pituitary adenoma Coronal T2-weighted image (A) and early dynamic contrast-enhanced T1-weighted image (B) show heterogenous lesion in the right half of the pituitary gland (~9 mm), showing central non-enhancing T2 hyperintense area and eccentric nodular enhancement (hypoenhancement relative to the rest of the pituitary gland), likely pituitary microadenoma.

For evaluation of pituitary disease, she underwent an MRI sella that revealed a 9-mm pituitary adenoma (Figures 5A, 5B).

She underwent removal of all parathyroid glands, cervical thymectomy, and auto-transplantation of normal parathyroid tissue into left forearm. Postoperatively, she was managed with calcium supplements and calcitriol. She also underwent total pancreatico-duodenectomy along with splenectomy. Post-surgery, glycemic control was achieved with insulin. Exocrine functions were managed with pancreatic enzyme supplements. For prolactinoma, she was started on tablet cabergoline 0.5 mg twice a week.

Histopathology of parathyroid specimens revealed parathyroid hyperplasia, while that of pancreatic specimen revealed well-differentiated neuroendocrine neoplasm, grade 1 (G1), with multiple multicentric microadenomas (pathological stage: pT2N0). Patient was counseled regarding the importance of regular follow-up. Investigations and their intervals were planned as defined for MEN1 patients [4].

Her brother, who was a 42-year-old male, also had symptoms suggestive of spontaneous hypoglycemia corrected by intake of carbohydrate diet and acromegaloid habitus. He too was admitted for evaluation and was found to have acromegaly with pituitary macroadenoma, primary hyperparathyroidism with adenomas in all the four parathyroid glands, and hyperinsulinemic hypoglycemia with multiple insulinomas. He too underwent total parathyroidectomy along with auto-transplantation of normal parathyroid tissue in non-dominant forearm, with cervical thymectomy. For pituitary macroadenoma, he underwent trans-nasal trans-sphenoidal surgery (TNTS) with uneventful recovery. However, he refused to undergo pancreatic surgery and instead resorted to medical therapy with frequent, low carbohydrate meals.

## Discussion

MEN1 is usually inherited as an autosomal dominant condition with a high degree of penetrance but can also be sporadic [5]. MEN1 can be diagnosed clinically when a patient presents with two or more MEN1-associated tumors (parathyroid adenomas, entero-pancreatic tumors, and pituitary adenomas). Diagnosis can be familial when a patient has a MEN1-associated tumor and a first degree relative with MEN1 [6]. Diagnosis can also be genetic when a person has MEN1 mutation without evident clinical or biochemical characteristics [6]. Commonly penetrated tumors in MEN1 are parathyroid adenomas (90%), entero-pancreatic tumors (30%-70%), and pituitary adenomas (30%-40%).

In our patient, the presenting complaint was that of hyperinsulinemic hypoglycemia, which was evaluated and diagnosed to be a case of multiple insulinomas. However, on detailed history she also had complaints directing toward involvement of other endocrine glands. Subsequently, she was evaluated and found to have primary hyperparathyroidism (PHPT) and prolactinoma. This case underlines the importance to look for symptomatology or biochemical clues suggestive of MEN1 syndrome when the patient presents with one of the MEN1-associated endocrine tumors. She, however, did not have other traditional hallmarks of MEN1 such as lipoma, collagenoma, angiofibroma, foregut carcinoid, or adrenal lesion.

Pre-operative imaging of pancreatic NET (neuro-endocrine tumor) not only aids in the diagnosis but also improves the success rate of surgery. Our patient underwent a structural imaging (triple-phase CT abdomen) as well as a functional imaging (Ga-68-DOTANOC study) for the localization of insulinoma. However, in MEN1, concurrent pancreatic NETs pose a challenge for the correct localization of the insulinoma. Endoscopic ultrasound remains a valuable pre-operative diagnostic tool for detection of pancreatic tumors, even up to 1-2 mm, which are otherwise undetectable by CT/MRI [7]. It is also challenging to differentiate between functioning and non-functioning pancreatic NET. Selective intra-arterial calcium stimulation test with hepatic venous gradient for insulin and C peptide can facilitate precise localization of insulinomas and can help differentiate between functioning and non-functioning pancreatic tumors [8]. In our patient, due to concomitant hypercalcemia, she was not subjected to intra-arterial calcium stimulation test.

While evaluating for PTH-dependent hypercalcemia, technetium-99m SESTAMIBI scan revealed adenomas in three parathyroid glands. On the contrary, 4D CT and 4D MRI revealed adenomas in all four glands. This does highlight the pitfall of technetium-99m SESTAMIBI scan as it provides false negative results in case of parathyroid hyperplasia or multiple parathyroid adenomas [9]. In MEN1 syndrome, pre-operative localization of parathyroid lesion is of limited benefit, and surgical exploration would be required irrespective of pre-operative imaging [4]. There has been controversy regarding the most preferred surgical approach of PHPT in MEN1. A few surgeons prefer subtotal parathyroidectomy, while others advocate total parathyroidectomy with auto-transplantation of normal parathyroid tissue into non-dominant forearm. Cervical thymectomy is also recommended along with parathyroidectomy in MEN1. Our patient had involvement of all four glands, hence was subjected to the latter surgical procedure. Measurement of intraoperative PTH by Turbo PTH assay and fulfillment of Miami criterion (more than 50% decline in serum PTH from the pre-incision value at 10 minutes post excision) [10,11] can help provide an idea about the immediate success of surgery.

MRI of the sella turcica revealed pituitary adenoma in our patient. Prolactinomas are the most common pituitary tumors in MEN1, and the same was observed in our patient, corroborating with clinical and biochemical presentation.

Clinical diagnosis of MEN1 was confirmed by genetic analysis in our patient. MEN1 gene on chromosome 11q13 encodes a 610-amino acid protein termed menin. Menin mediates stimulatory or inhibitory effects on numerous transcription factors by epigenetic mechanisms. Menin may act as a tumor suppressor or an oncogene depending upon its interaction with different proteins. Many proteins involved in DNA damage-dependent cell cycle arrest or subsequent DNA damage repair are dependent on the co-presence of functional menin [12].

Diagnosis of MEN1 and further confirmation by genetic analysis have many implications. MEN1-associated tumors differ from non-syndromic tumors in various aspects that are enlisted in Tables 1-3. These differences along with the need for comprehensive management and surveillance in MEN1 signify the importance of management of these patients by a multidisciplinary team.

**Table 1:** Differences between PHPT in MEN1 and non-MEN1 (sporadic) PHPT, primary hyperparathyroidism; MEN1, multiple endocrine neoplasia type 1; PTH, parathyroid hormone; BMD, bone mineral density.

	MEN1	Non-MEN1
Age of onset (years)	25	55
Female: male ratio	1:1	3:1
Biochemistry	Borderline rather than elevated PTH levels Mildly elevated serum calcium	Elevated PTH and calcium levels
Reduction in BMD	Greater	Lesser
Pathology	Multiglandular hyperplasia	Solitary adenoma
Surgery	Subtotal parathyroidectomy (at least 3.5 glands) or total parathyroidectomy with auto-transplantation. Concurrent transcervical thymectomy	Surgical removal of the abnormal gland
Intraoperative PTH	More useful	Useful
Post-operative rate of hypoparathyroidism	Higher	Lower
Recurrence rates	High	Low
Parathyroid cancer	Almost never progresses to parathyroid cancer	Might

**Table 2:** Differences between entero-pancreatic tumors in MEN1 and non-MEN1 (sporadic) MEN1, multiple endocrine neoplasia type 1.

	MEN1	Non-MEN1
Age at diagnosis (years)	10–50	50–80
Multiplicity of lesions	Frequently appear in multiples and occur on a background of diffuse micro-adenomatosis	Usually solitary pancreatic lesions
Most common functional pancreatico-duodenal neuroendocrine tumor	Gastrinoma	Insulinoma
Gastrinoma	Usually located in the duodenum and are small and multicentric	Larger and mostly singular, commonly arise from within the pancreas
Treatment of gastrinoma	Majority managed by proton pump inhibitors (as they are multicentric)	Majority undergo surgical exploration with curative intent

**Table 3:** Differences between pituitary tumors in MEN1 and non-MEN1 (sporadic) MEN1, multiple endocrine neoplasia type 1.

	MEN1	Non-MEN1
Macroadenomas	85%	42%
Incidence of multiple hormone expression and multiple adenomas	Higher	Lower
Aggressiveness	More	Less
Rate of hormonal control	Worse (42%)	Better (90%)
Treatment	Same in both groups	

## Conclusions

We have described a case of multiple endocrine neoplasia type 1 (MEN1) syndrome who presented to us with episodes of spontaneous hypoglycemia. The diagnosis of MEN1 syndrome in our patient led to the discovery of a large kindred being affected with the same disease. This underlies the importance for searching for MEN1-related tumors and genetic screening in family members on the basis of clinical and biochemical suspicion.
